# Charge Isomers of Myelin Basic Protein: Structure and Interactions with Membranes, Nucleotide Analogues, and Calmodulin

**DOI:** 10.1371/journal.pone.0019915

**Published:** 2011-05-25

**Authors:** Chaozhan Wang, Ute Neugebauer, Jochen Bürck, Matti Myllykoski, Peter Baumgärtel, Jürgen Popp, Petri Kursula

**Affiliations:** 1 Department of Biochemistry, University of Oulu, Oulu, Finland; 2 Institute of Photonic Technology, Jena, Germany; 3 Institute for Biological Interfaces 2, Karlsruhe Institute of Technology, Karlsruhe, Germany; 4 Berlin Electron Storage Ring Company for Synchrotron Radiation, Helmholtz-Zentrum Berlin, Germany; 5 Institute for Physical Chemistry, Friedrich-Schiller-University Jena, Jena, Germany; 6 Centre for Structural Systems Biology, German Electron Synchrotron, Hamburg, Germany; University of Oldenburg, Germany

## Abstract

As an essential structural protein required for tight compaction of the central nervous system myelin sheath, myelin basic protein (MBP) is one of the candidate autoantigens of the human inflammatory demyelinating disease multiple sclerosis, which is characterized by the active degradation of the myelin sheath. In this work, recombinant murine analogues of the natural C1 and C8 charge components (rmC1 and rmC8), two isoforms of the classic 18.5-kDa MBP, were used as model proteins to get insights into the structure and function of the charge isomers. Various biochemical and biophysical methods such as size exclusion chromatography, calorimetry, surface plasmon resonance, small angle X-ray and neutron scattering, Raman and fluorescence spectroscopy, and conventional as well as synchrotron radiation circular dichroism were used to investigate differences between these two isoforms, both from the structural point of view, and regarding interactions with ligands, including calmodulin (CaM), various detergents, nucleotide analogues, and lipids. Overall, our results provide further proof that rmC8 is deficient both in structure and especially in function, when compared to rmC1. While the CaM binding properties of the two forms are very similar, their interactions with membrane mimics are different. CaM can be used to remove MBP from immobilized lipid monolayers made of synthetic lipids - a phenomenon, which may be of relevance for MBP function and its regulation. Furthermore, using fluorescently labelled nucleotides, we observed binding of ATP and GTP, but not AMP, by MBP; the binding of nucleoside triphosphates was inhibited by the presence of CaM. Together, our results provide important further data on the interactions between MBP and its ligands, and on the differences in the structure and function between MBP charge isomers.

## Introduction

The presence of the myelin sheath, a tightly packed multilamellar membrane, is crucial to the functioning of the vertebrate nervous system. Myelin is formed by specialized glial cells in both the central and peripheral nervous systems (CNS and PNS, respectively), and mutations in myelin components or autoimmune attack towards them leads to severe neurological defects. Many of the defects observed in dys- or demyelination can be attributed to potential disruption of the intimate interactions between myelin proteins and the myelin lipid bilayer.

The myelin basic protein (MBP) is one of the most abundant proteins in myelin, and present at high concentration in both the CNS and PNS myelin [1], [2]. MBP is a peripheral membrane protein, which is reminiscent of intrinsically disordered proteins, when put into aqueous solution [3]–[5]. Several lines of evidence, however, point towards a scenario, where interactions with ligands trigger secondary structure formation and some degree of folding into a more compact structure in MBP [6]. Previously, we have carried out detailed structural analyses on the calmodulin (CaM) complexes of full-length MBP and peptides from the C-terminal CaM interaction site of MBP [7], [8].

MBP has a multitude of isoforms generated by both complex alternative splicing and a wealth of post-translational modifications [9]. In the nervous system, it is present as different charge isomers, and it is evident that charge effects modulate its function and interactions with ligands, such as membrane surfaces [10]–[13]. MBP is one of the main autoantigens of the myelin sheath, being implicated in multiple sclerosis (MS) and animal models of autoimmune neurological disorders [14], [15]. Post-translational modifications of MBP may play an important role for in pathogenesis of MS [16]. Deimination of arginine occurs at a number of sites and was elevated in MS [16], and the degree of deimination (or citrullination) of MBP is correlated with the severity of MS [17].

Previously, recombinant C1 and C8 isomers (rmC1 and rmC8) of mouse 18.5-kDa MBP (rmMBP) have been used to obtain significant insights into the structure-function relationships of MBP and the likely effects of charge-neutralizing post-translational modifications, such as those seen in the C8 isoform [18]. The recombinant rmC8 form is based on the charge isomer C8 of MBP detected at high levels *e.g.* in the aggressive Marburg variant of MS [19]. In rmC8, six residues of murine 18.5-kDa MBP isoform have been replaced by a glutamine residue: Arg 23, 31, 127, 157, and 168, as well as Lys 119 [19]. The pseudodeiminated rmC8 form has, for example, been studied with respect to membrane interactions [11] and actin polymerization [20], [21]. We have also recently conducted a study, using atomic force microscopy, where it was observed that while both rmC1 and rmC8 bound to supported lipid bilayers, only rmC1 was able to effectively induce the spontaneous stacking of these bilayers on top of each other [22]. Whether the functional differences between rmC1 and rmC8 are caused by differences in structure, function, or both, is still not completely clear.

In the current study, we have used several biophysical techniques to further characterize the recombinant MBP isoforms rmC1 and rmC8, including interactions with different types of ligands and structural analyses. The results indicate that while both isoforms have similar properties, in general, the rmC8 form is impaired in both ligand binding and structure.

## Materials and Methods

### Protein purification

The recombinant MBP isoforms were purified essentially as previously described, using Ni-NTA under denaturing conditions [19], [22], [23]. Recombinant CaM was expressed and purified as described earlier [7], [24], using calcium-dependent chromatography on phenyl sepharose.

### Special materials

All lipids (dimyristoylphosphatidylcholine (DMPC), dimyristoylphosphatidylglycerol (DMPG), phosphatidylcholine (PC), phosphatidylinositol (PI), phosphatidylinositol-4-phosphate (PIP), and phosphatidylinositol-4,5-bisphosphate (PIP_2_)) were purchased from Avanti Polar Lipids. The phosphocholine detergents (with alkyl chain lengths between 8–12 carbons) and n-dodecyl-N,N-dimethylamine-N-oxide (LDAO) were from Anatrace.

### Small-angle X-ray scattering

Synchrotron small-angle X-ray scattering (SAXS) data for rmMBP were collected on beamline I711 [25] at MAX-Lab, Lund, Sweden, and analyzed as previously described [7], [8], [26]. An exposure time of 10 min was used for all samples. The ATSAS package [27] was used for data processing and further data analysis and molecular modeling. In particular, GNOM [28] was used for generating the distance distribution function; *ab initio* models of the samples were obtained using the program GASBOR [29], which represents the model as a chain-like assembly of dummy residues. The final model was obtained in each case from 10 independent *ab initio* models by averaging by DAMAVER [30]. CaM was used as a molecular weight standard due to its similar size to MBP and previously observed good behaviour in our SAXS experiments [7], [26].

### Small-angle neutron scattering

For small-angle neutron scattering (SANS) experiments, rmC1 and rmC8 were dialyzed against 4 mM HEPES, pH 7.5, 20 mM NaCl, in 98% D_2_O. SANS data were collected on beamline SANS1 at GKSS (Geesthacht, Germany), and were processed on absolute scale.

### Synchrotron radiation CD spectroscopy

Synchrotron radiation circular dichroism (SRCD) spectroscopy data from solution samples, including samples measured in membrane-mimetic conditions, were collected on beamline CD1 at ISA, University of Århus, Denmark, as previously described [31]. Samples were scanned from 280 nm to 170 nm, in steps of 1 nm. The absolute CD sensitivity was verified by measuring the calibration standard (+)-10-camphorsulfonic acid [32]. A circular quartz cuvette with a pathlength of 18 or 100 µm was used, depending on the protein concentration. Data processing and analysis were carried out in CDtool [33]. Each spectrum is the baseline corrected average of 3 successively executed scans of the sample. The measurements in detergent solutions had protein concentrations of 0.25 mM, and the spectra with TFE were measured with 0.05 mM protein. In addition, MBP samples were dried as films on a calcium fluoride cuvette, and dry phase SRCD spectra were collected at the 3m-NIM-C beamline [34] at BESSY, Berlin, as previously described [31], [35]. For more accurate comparison, the dry phase spectra were corrected for protein concentration by dividing with the absorbance at 190 nm.

### CD spectroscopy in the presence of lipid vesicles

First, multilamellar vesicles (MLV) were prepared by dissolving DMPC and DMPG in a 1∶1 ratio in CHCl_3_/MeOH, removing the organic solvents under dry nitrogen and 3 h in vacuum, and suspending them into 10 mM phosphate buffer at 1.46 mM concentration. Small unilamellar vesicles were formed by sonication of the MLVs for 5 min. rmC1 was dialyzed into 10 mM potassium phosphate, pH 7. After mixing MBP with the vesicles, the samples were further sonicated to prevent extensive aggregation. CD spectra were collected on a Jasco J-815 spectropolarimeter, and the ratio of protein to lipid was screened until the effect of absorption flattening was considered to be insignificant. In the end, good spectra could be measured at a protein/lipid molar ratio of 1/300, corresponding to a protein concentration of 95.5 µg/ml. CD spectra for vesicle-free rmC1 were similarly measured in phosphate buffer. Secondary structure content was estimated using CDSSTR [36] at Dichroweb [37].

### Analysis of MBP conformation by Raman spectroscopy

Further conformational analyses were performed by Raman spectroscopy using a HR LabRam Raman spectrometer (Horiba Jobin-Yvon) with a focal length of 800 mm. The recombinant MBP was dialyzed into 10 mM potassium phosphate buffer (pH 7.0), and frozen in small aliquots. The samples were lyophilized and the lyophilized protein placed under a microscope (Olympus). Raman spectra were excited with a 532 nm laser (SUWTECH LDC-1500, Shanghai, China), which was focussed on the sample through a 40× microscope objective (Olympus, NA 0.75). Laser power behind the objective was 4.8 mW. The Raman scattered light was collected in 180° back-scattering and recorded on a liquid nitrogen cooled CCD camera (1024×512 pixels). For overview spectra, a 300 lines/mm grating (spectral resolution 6.3 cm^−1^) was used. A close-up of the fingerprint region was recorded using a grating of 1800 lines/mm (spectral resolution 1.9 cm^−1^). The fingerprint Raman spectra were background corrected using the sensitive nonlinear iterative peak (SNIP) clipping algorithm [38] implemented in gnu R [39] and normalized in three regions prior to computing the difference spectrum.

### Surface plasmon resonance

All the interaction analyses studied by surface plasmon resonance (SPR) were conducted on a Biacore 3000 system (GE Healthcare) at +25°C. After the run, the data were analyzed using the BIAevaluation software or GraphPad Prism to obtain K_d_ values for the interactions.

For binding between rmMBP and CaM, CaM was covalently immobilized onto a CM5 chip (GE Healthcare) using standard amine-coupling chemistry, essentially as previously described [7], [8]. One flow path without CaM was used as a reference throughout the experiment. A flow rate of 10 µl/min was used during the run; various concentrations of rmMBP (0.01–1 µM) in running buffer (20 mM HEPES (pH 7.5), 100 mM NaCl, and 10 mM CaCl_2_) were injected over both of the flow cells for 3 min, followed by a 6-min dissociation phase after each injection. The surfaces were regenerated with a 3-min injection of 200 mM EDTA and another 3-min injection of 1 M NaCl prior to the next injection cycle. All experiments were performed in duplicate. In a reverse experiment, immobilization of MBP on an NTA chip (GE Healthcare) through the C-terminal His-tag was carried out according to the manufacturer's instructions, and CaM binding was assessed as above.

For binding between rmMBP and lipid mono- or bilayers, homogeneous unilamellar lipid vesicles were made with different lipid compositions. Lyophilized lipids were dissolved in chloroform, mixed in pre-defined ratios when appropriate, and the solvent was removed by evaporation under nitrogen. Lipid films were hydrated at room temperature to a concentration of 5 mM, in a buffer containing 20 mM HEPES (pH 7.5) and 100 mM NaCl, which was also used as the running buffer. The lipid suspensions were subjected to five cycles of freezing (liquid nitrogen), thawing (+40°C), and vortexing (5 s). Then, the suspensions were extruded 15 times through a 100-nm polycarbonate membrane, using an Avanti Mini-Extruder (Avanti Polar Lipids, Alabaster, AL, USA). Supported PC, PC/PI (9∶1), PC/PIP (9∶1), and PC/PIP_2_ (9∶1) surfaces were formed on HPA or L1 chips (GE Healthcare) by incubating unilamellar vesicles at a total lipid concentration of 0.5 mM in the running buffer for 3 h at 25°C at 5 µl/min, following the instructions of the chip manufacturer. A 5-min injection of 0.1 mg/ml BSA indicated practically no unspecific binding on the coated chips. A flow rate of 5 µl/min was used during the run, in which various concentrations of rmMBP (0.01–2 µM) were injected in duplicate over the flow cell for 6 min, followed by a 6-min dissociation phase after each injection. The surfaces were regenerated with a 6-min injection of 50 mM NaOH prior to the next injection cycle.

For analyzing the effect of Zn^2+^ on the binding of rmMBP on supported lipids, 1 µM rmMBP containing various concentrations of zinc acetate (0–10 mM) was injected in duplicate over the flow cell (containing immobilized PC on a HPA chip) for 6 min, while 20 mM HEPES (pH 7.5) containing 100 mM NaCl was used as the running buffer. The subsequent procedures were as above.

For the analysis of the effect of CaM on the binding of rmMBP on the supported lipid surface, 20 mM HEPES (pH 7.5) containing 100 mM NaCl, and 10 mM CaCl_2_ was used as the running buffer. 1 µM rmMBP prepared in the running buffer was first injected over the flow cell containing immobilized PC for 6 min, then various concentrations of CaM (0–50 µM) were injected over the flow cell for 6 min. The subsequent procedures were as mentioned above, except that a 6-min injection of 0.2 M EDTA was performed prior to the 6-min injection of 50 mM NaOH during regeneration. 1 µM MBP was used, since it was observed in PC monolayer binding experiments that both rmC1 and rmC8 caused a saturation binding already below 1 µM (see Results). The amount of MBP removed by the CaM injection was obtained by subtracting from the resonance signal right before the CaM injection the signal a few seconds after the end of the CaM injection. An injection without CaM was used to assign the amount removed spontaneously during the injection period.

### Size exclusion chromatography

Mixtures with different ratios of rmMBP and CaM were used to analyze protein complex formation by size exclusion chromatography. A Superdex 200 HR 10/30 column and an ÄKTA *purifier* system (GE Healthcare) were used; a buffer containing 20 mM HEPES pH 7.5, 100 mM NaCl, and 10 mM CaCl_2_ was the mobile phase. The flow rate was 0.3 ml/min, and the UV detection wavelength was set at 280 nm. The protein concentration was 50 µM for both rmMBP and CaM alone; for the complexes, the concentration of rmMBP was fixed at 25 µM, and the concentration of CaM was changed from 12.5 µM to 125 µM for different rmMBP∶CaM ratios (2∶1, 1∶1, 1∶2, 1∶3, and 1∶5). The loading volume for all samples was 500 µl. All chromatographic analyses were carried out at room temperature.

### Isothermal titration calorimetry

Isothermal titration calorimetry (ITC) was used to analyze the binding of CaM to rmMBP. Prior to the ITC measurements, CaM and rmMBP were extensively dialyzed against 20 mM HEPES (pH 7.5), 100 mM NaCl, 10 mM CaCl_2_ at +4°C. rmMBP and CaM were diluted with the dialysis buffer to 5 µM and 100 µM, respectively, and degassed in MicroCal ThermoVac for 8 min at a temperature 2–4°C lower than the titration temperature. rmMBP was loaded into the measurement cell of the MicroCal VP-ITC calorimeter (Microcal Inc) and titrated with 28 10-µl injections of CaM (in the syringe). The experiment was carried out at +15, +20, +25 and +30°C. The data were analyzed by integrating the peak areas in MicroCal Origin (MicroCal) and fitted by non-linear least squares minimization method using a model for one binding site (Levenberg-Marquardt algorithm). Our previous ITC data with MBP isolated from brain had indicated clearly the presence of two distinct binding sites [8], but the two binding sites were less clearly observed for the recombinant MBP in the current study.

### Analysis of fluorescent nucleotide binding

In initial experiments, we observed that MBP bound fluorescent ATP and GTP analogues (unpublished data). Here, we further studied this binding and the effect of CaM therein by fluorescence spectroscopy, using a Tecan Infinite M200 apparatus, in black flat-bottom 96-well plates. The assay was performed using N-methyl-anthraniloyl-labelled (mant) nucleotides (mant-ATP, mant-AMP, and mant-GTP; Jena Bioscience). A mixture of rmC1 (0.25 µM) and mant nucleotides (0.5–12.5 µM) was made in 10 mM HEPES buffer, pH 7.5, in a total volume of 200 µl. For testing the effect of CaM, 0.5 mM CaCl_2_ and 7.5 µM CaM were included. This assay used FRET *via* Trp fluorescence excitation of mant fluorescence. Thus, the excitation was at 280 nm and emission was collected between 310–500 nm, with the peak of mant fluorescence expected at 448 nm. As a control, mant was also directly excited at 355 nm, and emission measured between 400–500 nm.

A mixture of MBP (rmC1, 1.3 µM; rmC8, 0.8 µM) and O-trinitrophenyl-labelled (TNP) ATP (Jena Bioscience) (5 µM) was made in 20 mM HEPES buffer, pH 7.5, containing 2 mM CaCl_2_, and CaM was included as a concentration series of 75 nM - 2 µM. A control series was also prepared without MBP, and all samples were prepared in duplicate. Fluorescence emission spectra between 510 and 600 nm were collected using an excitation wavelength of 470 nm, at +25°C.

### Intrinsic Trp fluorescence analysis on dodecylphosphocholine binding

The effect of dodecylphosphocholine (DPC) on intrisic Trp fluorescence emission was checked by fluorescence spectroscopy for both rmC1 and rmC8, essentially as described previously [31]. The proteins (rmC1, 1.3 µM; rmC8, 0.8 µM) were diluted into 10 mM KPO_4_ (pH 7) with 0–2% DPC (0–57 mM). Excitation was done at 280 nm and fluorescence emission spectra were collected between 320–400 nm. The experiment was carried out at +25, +30, +37, and +42°C. The observed fluorescence emission was corrected for the difference in protein concentration.

## Results and Discussion

### 3D solution structure of rmMBP

**Figure 1:**
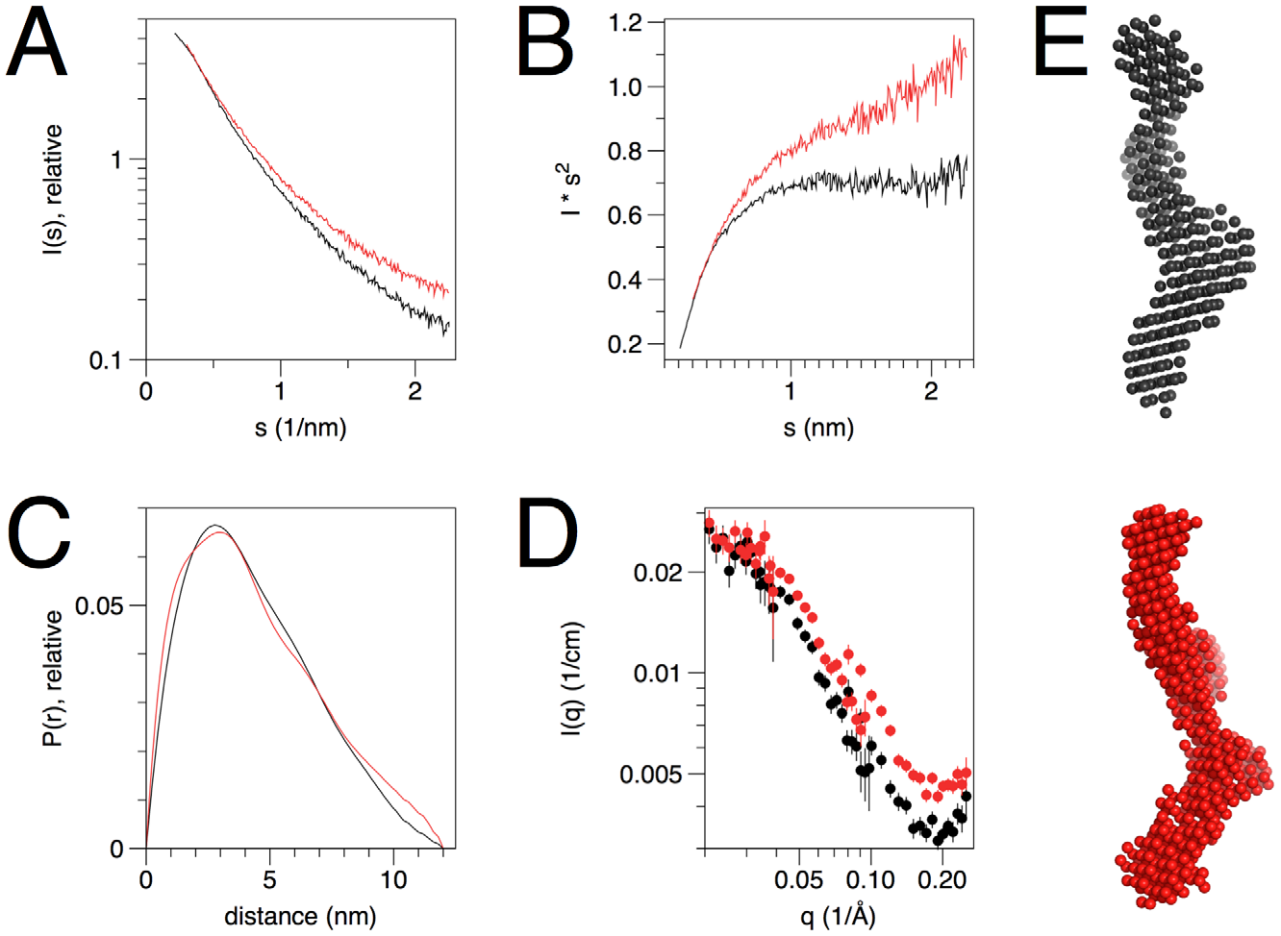
Conformation of recombinant MBP. In all figures, data from rmC1 is coloured black and from rmC8 red. A. X-ray scattering curves for rmC1 and rmC8. B. Kratky plots, indicating the absence of globular folded domains. C. Distance distribution function indicates a maximum dimension of 12 nm for both MBP isoforms. D. SANS scattering curves for rmC1 and rmC8. The data are on absolute scale, and both axes are logarithmic. E. *Ab initio* models showing unfolded molecules with a maximum dimension of 12 nm.

As an overall test for folding of the rmC1 and rmC8 forms of MBP in solution, synchrotron SAXS experiments were carried out (Figure 1, Table 1). Both variants had a tendency to aggregate during X-ray exposure (data not shown), and thus, only single scattering experiments with the proteins alone are presented here. As expected [5], [8], [40], both proteins present X-ray scattering compatible with an intrinsically disordered molecule, with a highly extended shape and a maximum dimension of approximately 12 nm. Similar results were also obtained with SANS, where the neutron scattering curve indicates a random coil structure (Figure 1D). The result indicates that both forms behave in solution essentially similarly to the protein isolated from nervous tissue and freed from lipid contaminants [8], [41], and that there are no large conformational differences between rmC1 and rmC8 in aqueous solution.

**Table 1:** Results from SAXS analysis.

Sample	I(0), relative	R_g_, nm	Volume of *ab initio* model, (nm^3^)	D_max_, nm	MW(kDa)^a^	MW(kDa)^b^	MW(kDa)^c^
CaM	5.08	2.06	29.2	7.0	17	-	-
rmC1	5.13	3.51	34.0	12	19	17.2	19.8
rmC8	5.31	3.63	34.0	12	19	17.8	19.8

CaM was used as a reference sample.

a calculated from sequence.

b calculated from I(0), comparing to CaM.

c calculated from volume, comparing to CaM.

### Fine structural details from Raman spectroscopy

Raman spectroscopy is based on the inelastic scattering of monochromatic radiation (laser light). When a sample is irradiated, energy is exchanged between the excitation light and the molecular vibrations of the investigated molecules. This results in a measurable shift in the wavelength of the incident light which is represented in the Raman spectrum. The Raman band positions and intensities are determined by the types of atoms (C, O, N, H, S) connected in the molecule, how they are linked (single bond, double bond, triple bond), and which local environment they experience (*e.g.* hydrogen bonding). Therefore, the observed Raman spectra are like “molecular fingerprints”, containing information about covalent structure, conformation, and local environment of the functional groups.

**Figure 2:**
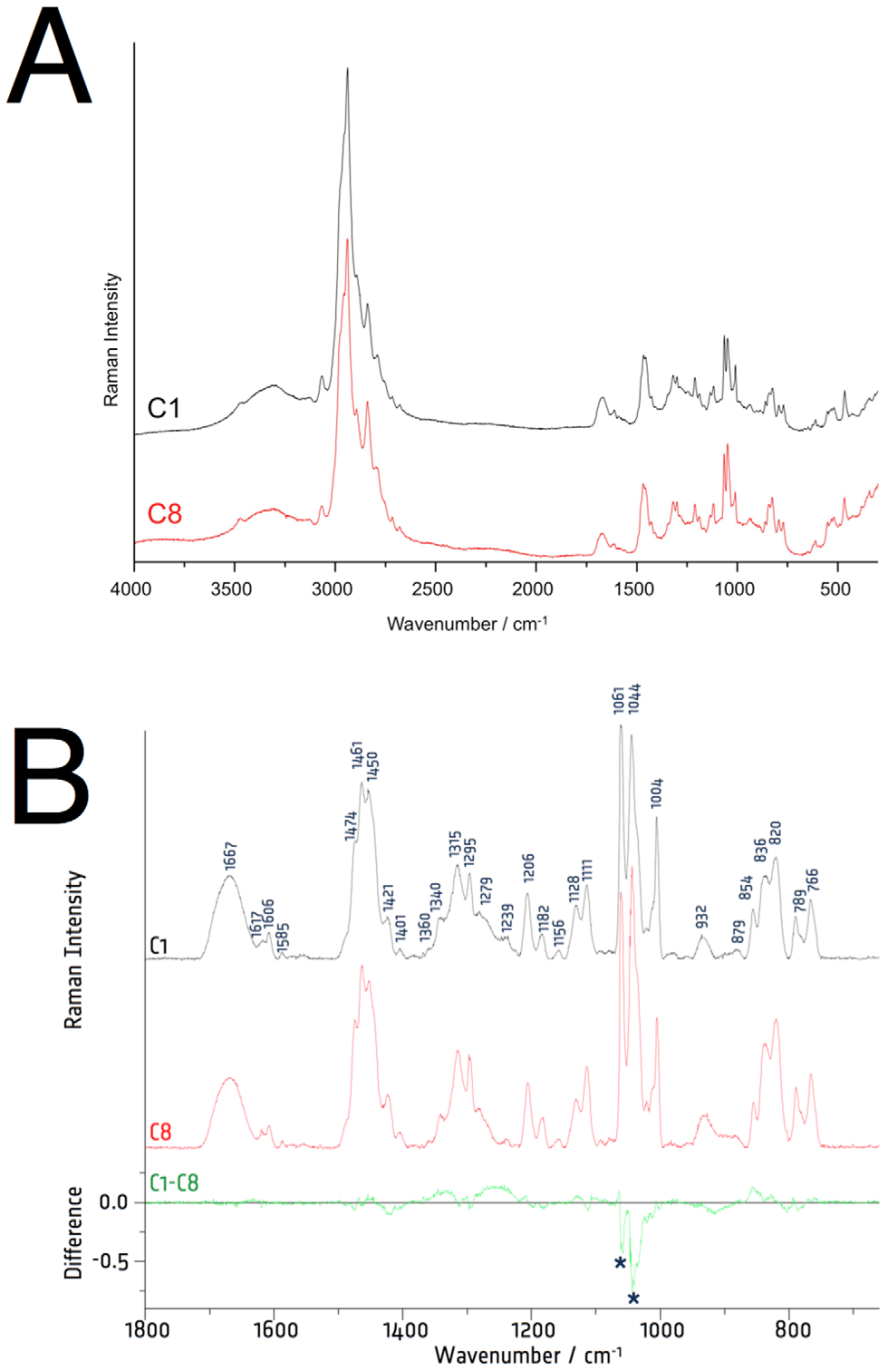
Raman spectroscopy (rmC1 – black; rmC8 – red). A. Raman spectra of the rmC1 and rmC8 isoforms of MBP in the spectral region of 4000 – 200 cm^−1^. B. A close-up of the fingerprint region (1850-660 cm^−1^) of the same samples.

As a further test of potential structural differences between rmC1 and rmC8, we carried out Raman spectroscopy of lyophilized samples. Both samples behaved essentially identical, showing a number of common features, as can be seen in Figure 2A, showing the full spectral region (4000 – 200 cm^−1^), and in the fingerprint region (1850-660 cm^−1^) (Figure 2B). A Raman difference spectrum was also computed by subtracting the Raman spectrum of rmC8 from rmC1, using background corrected and normalized spectra, in order to highlight spectral differences. A detailed assignment of the vibrational bands, based on data from the literature, is given in Table 2.

**Table 2:** Assignment of Raman spectroscopic bands.

Wavenumber (cm^−1^)	Assignment	Reference
1667	amide I	[65]
1617	W (W1)/Y (Y8a)	[65]/[66]
1606	F (F8a)	[65]
1585	F (F8b), W (W2)	[65]
1474	CH_2_ bend	[65], [66], [67]
1461	CH_3_ bend, aliphat CH_2_ def, W (W5), F	[65]/[66], [68]
1450	K, I, L, aliph. CH_2_ def	[68], [67]
1421	W (W6)	[65]
1401	CO_2_^−^ str	[67]
1357	W (W7″), V, F, CH bend	[68], [65]
1339	W (W7′), amide III	[67], [42]
1315	aliph CH_2_ def/CH bend	[66], [67]/[65]
1230–1290	amide III	[67], [69]
1206	F (F7a), Y, W (W11?)	[68], [67], [65]
1182	Y (Y9a), F	[66] (1179), [68] (1179)
1156	I, V, CH_3_ def	[68], [67]
1128	I, V, L, W (W13), aliph. CH3 def	[68], [67], [42]
1117	A, C-N str.	[68], [69]
1061	K, A, F/C-C str/* possible matrix contributions	[68] (1056)/[66]
1044	T/C-C str/* possible matrix contributions	[65]/[66]
1034	F (F18a), Y	[66] (1030), [68] (1031)
1011	W (W16)	[68] (1010), [67], [42], [66]
1004	F (F12)	[68], [67], [66], [65]
935	K, V, L	[68]
879	W (W17)/K	[66], [67], [42]/[68] (885)
854	Y (Y1+Y16a), I/CCN str, bend	[66], [68] (853)/[65]
836	Y (Y1+Y16a)	[66] (829)
820	F (F1)	[65] (827)
789		
766	CH rock, skeletal/W (W18)	[68]/[65]

Used abbreviations: amino acids one letter code; types of vibration: bend - bending, def - deformation, str - stretching vibration, rock - rocking, tor - torsion.

The prominent feature between 3000 and 2700 cm^−1^ represents the CH, CH_2_ and CH_3_ stretching vibrations, and the broader band at higher wavenumbers is due to OH and NH stretching vibrations. The amide I band is found around 1666 cm^−1^. This vibrational band is due to the C = O stretching mode of the peptide linkage. Depending on the secondary structure, the exact wavenumber position can change between 1640 and 1680 cm^−1^. If hydrogen bonds are formed between the C = O and NH of different peptide backbone chains, the amide I band can be found around 1670 cm^−1^ [42]. The position and shape of the amide I band look nearly identical for both rmC1 and rmC8, and in the difference spectrum only minute differences are visible. Therefore, it can be concluded that there is no significant change in the protein secondary structure. It is possible that significant amounts of secondary structure are present in the samples when lyophilized; this is supported by the comparison of SRCD spectra in the solution and solid states (see below).

Aromatic rings are good Raman scatterers, and therefore, Raman bands characteristic for aromatic amino acids are quite strong and can be identified in the protein spectrum, even though they might overlap with amide bands and other side chain groups. Very characteristic is the ring mode of phenylalanine (F12) at 1004 cm^−1^. Other vibrations indicative to phenylalanine are at 1606 cm^−1^ (F8a), 1204 cm^−1^ (F7a), 819 cm^−1^ (F1), and 622 cm^−1^ (F6b). Tryptophan exhibits vibrational bands at 1617 cm^−1^ (W1), 1576 cm^−1^ (W2), 1421 cm^−1^ (W6) and the Fermi doublet at 1357 cm^−1^ (W7″) and 1339 cm^−1^ (W7′), respectively. The relative intensity of these two Fermi doublet bands is assumed to be a good indicator for the hydrophobicity of the tryptophan indole ring environment [43], [44]. Even though the latter bands are not so well resolved in the spectra, it can be estimated that the relative ratio of the intensities I_1360_/I_1340_ is smaller than 1, indicating that the indole ring is not strongly shielded by aliphatic side chains. The respective bands in the spectra of rmC1 and rmC8 are almost identical, which means no significant differences are present in the local environment of phenylalanine and tryptophan in the two charge isomers. In the computed Raman difference spectrum, a broad background feature makes the interpretation of this spectral region very difficult.

Tyrosine exhibits a Fermi doublet around 850 cm^−1^ (Y1+Y16a) and 830 cm^−1^ (Y1+Y16a). The intensity ratio of the two bands can be correlated to the hydrogen bonding states of the Tyr hydroxyl group [42]. For rmC1, the relative ratio I_854_/I_838_ seems to be slightly larger than for rmC8, as can be also seen in the positive feature in the computed difference spectrum. This indicates that in rmC8, the tyrosine might be a slightly stronger donor of hydrogen bonds than in rmC1. Further tyrosine Raman bands are found at 644 cm^−1^ (Y6b), 1182 cm^−1^ (Y9a) and 1617 cm^−1^ (Y8a), the latter one overlapping with the tryptophan band W1.

The Raman spectroscopy experiments indicate that the structures of rmC1 and rmC8 are essentially identical in the lyophilized samples. The single Trp residue (Trp113) is possibly solvent-exposed, and small differences might exist locally in the hydrogen bonding of the 5 Tyr residues. For example, Tyr125 is between two of the residues mutated to Gln in rmC8 (Lys119, Arg128). The position of the amide I band also suggests the presence of secondary structures, which may be induced by lyophilization.

### Effect of membrane-like conditions on secondary structure of MBP

**Figure 3:**
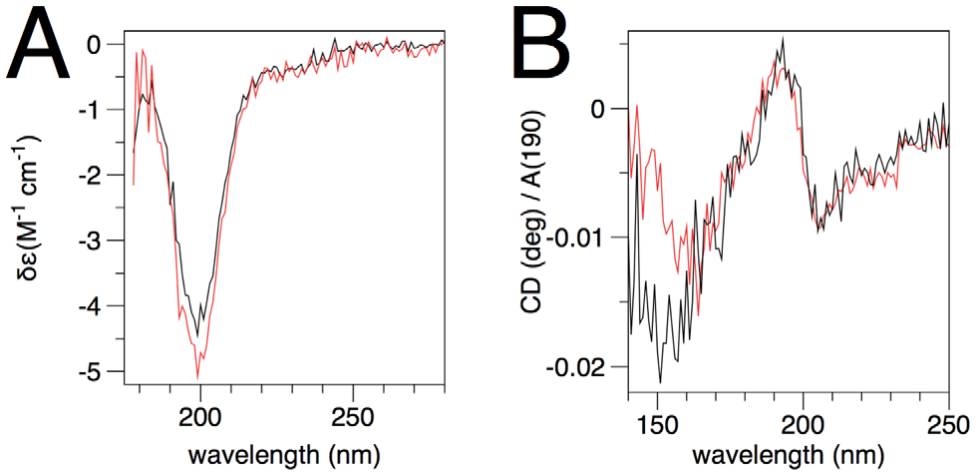
SRCD analysis of recombinant MBP isoforms. The spectra of the MBP isoforms were measured in solution (A) and as dried films (B). The spectra in (B) were corrected for protein amount by dividing with A(190). rmC1 – black; rmC8 – red.

It is well known that MBP has a disordered structure in aqueous solution [5], [40]. SRCD spectra of rmC1 and rmC8 show a strong negative peak at 199 nm and no positive peak (Figure 3A), which is a typical characteristic of an unfolded structure. There is no obvious difference between the two structures, as far as the CD signal is concerned. SRCD spectra were also measured from dried protein films as previously described [31], [35], down to 130 nm (Figure 3B). Differences can only be seen in the region below 160 nm, while both proteins obviously tend to fold into some degree of helical structure upon slow drying onto the cuvette surface. This phenomenon could be analogous to the partial folding observed in membrane-mimetic conditions (see below) and the position of the amide I band in Raman spectra (see above).

**Figure 4:**
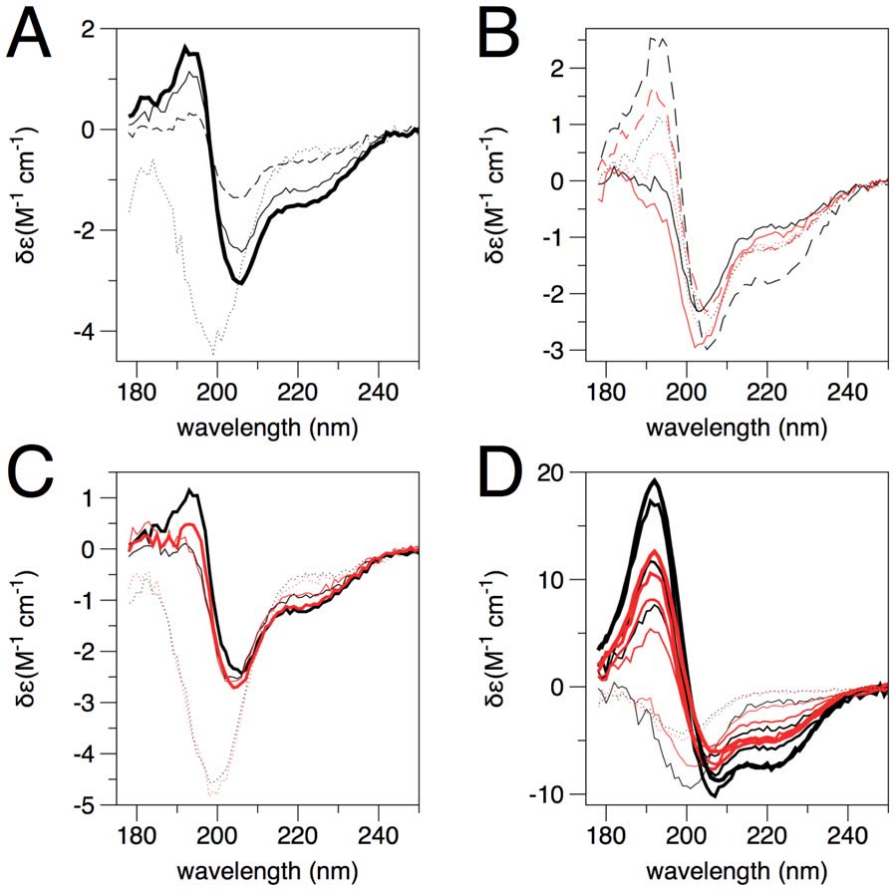
SRCD analysis of MBP in membrane-mimetic solution environment. A. Titration of rmC1 with DPC. Dotted line, phosphate buffer; dashed line, 25 mM DPC; thin line, 50 mM DPC; thick line, 100 mM DPC. B. Comparison of the effect of different detergent micelles on rmC1 (black) and rmC8 (red). Dotted line, 50 mM DPC; dashed line, 25 mM SDS; solid line, 50 mM LDAO. C. Comparison of 50 mM phosphocholines of different alkyl chain length on rmC1 (black) and rmC8 (red). Dotted line, octylphosphocholine; thin line, nonylphosphocholine; thick line, dodecylphosphocholine (DPC). D. TFE-induced folding of rmC1 (black) and rmC8 (red). The dotted lines are the spectra without TFE, and the solid lines from the thinnest to the thickest represent TFE concentrations 10, 30, 50, 70, and 90%.

**Figure 5:**
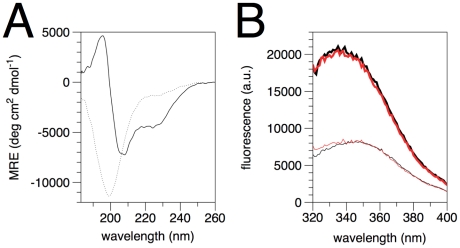
Features of MBP interaction with membranes and micelles. A. CD analysis of MBP conformation bound to lipid vesicles. Dotted line, MBP in phosphate buffer; solid line, MBP bound to DMPG/DMPC vesicles at a protein/lipid ratio of 1/300. MRE, mean residue ellipticity. B. The interaction with DPC micelles, as observed by intrinsic tryptophan fluorescence. Both rmC1 (black) and rmC8 (red) have increased Trp fluorescence in the presence (thick lines) of 0.1% DPC, compared to its absence (thin lines). The identical result was observed at temperatures between +25 and +42°C, and in the DPC concentration range 0.1–2.0%. The shift of the peak to a much lower wavelength while increasing its intensity is a sign that the single Trp residue of MBP is buried inside the DPC micelle, in a hydrophobic environment.

The effects of several membrane mimics, including DPC and other phosphocholines, LDAO, SDS, and TFE, on the secondary structure of MBP were investigated by SRCD (Figure 4). It was pointed out that DPC micelles can constitute a realistic model of membrane interfaces [45], and thus, we decided to study the effect of different concentrations of DPC on the structure of rmMBP. Such experiments were carried out already previously with rmC1 and conventional CD spectroscopy [46], at much lower protein concentration. With the addition of DPC, rmC1 acquired a significant fraction of helical secondary structure (Figure 4A). In addition, all the three distinctive helical peaks increased with increasing DPC concentration from 25 mM to 100 mM. The effect of DPC on rmC8 is similar to rmC1 (Figure 4B); however, rmC8 has a weaker propensity to obtain secondary structure in the presence of DPC. Changes in recombinant MBP conformation were, thus, induced by the interaction between the protein and the DPC detergent, as also seen before [47]. Trp fluorescence was also used to confirm binding to DPC micelles, and a saturation level of binding for both proteins was observed at 0.1% DPC (Figure 5B). Since only one Trp residue is present in MBP, the result indicates its environment changes upon micelle binding; most likely, it gets buried within the hydrophobic core of the micelle.

A series of phosphocholine derivatives were used to assess the effect of alkyl chain length on the capacity of the detergent to induce secondary structure in rmMBP (Figure 4C). Octylphosphocholine has hardly any effect on the structure of rmC1; the likely reason is that the binding only occurs between MBP and detergent micelles, and the concentration used here is lower than the critical micellar concentration (CMC) of octylphosphocholine, about 114 mM. On the other hand, nonyl-, decyl-, undecyl-, and dodecylphosphocholine induce secondary structure formation in rmC1, with two negative peaks approximately at 206 nm and 223 nm. For detergents with alkyl chains longer than 10 carbons, there is also an additional positive peak at 192–193 nm. All these data suggest folding into α-helical structure. rmC8 shows similar results, except that the positive peak at 192–193 nm is very weak also in DPC. Thus, rmC8 apparently has a somewhat weaker capacity to fold in the presence of alkylphosphocholine micelles (Figure 4C). The differential effects seen with different alkyl chain lengths probably reflect the fact that the CMCs of the used detergents are different; for example, 50 mM is actually below the CMC for octylphosphocholine.

The effect of SDS on the secondary structure of rmC1 and rmC8 was also similarly assessed (Figure 4B). The results are very similar to the effect of DPC, but even lower detergent concentrations cause larger CD signals for helical structure. As a detergent widely used for membrane protein purification and crystallization, the effect of LDAO on the structure of rmMBP was also tested (Figure 4B). Two negative peaks, at 204 nm and 222 nm, similar to those in DPC, appeared. However, there is no positive peak around 192 nm. These observations may be related to a requirement of a phosphate (or sulphate) group for efficient MBP binding to micelles, or the phosphate group in phosphocholines and sulphate group in SDS are easier to access than the amine oxide group in LDAO. Overall, the result highlights the importance of electrostatic and hydrophobic interactions in MBP-membrane binding.

As one of the most widely used organic solvents to mimic membrane environment, by lowering the dielectric constant of the solution, TFE was assessed for its capacity to induce rmMBP secondary structure (Figure 4D). When the concentration of TFE is increased, two negative peaks at 220 and 208 nm and one positive peak at 192 nm appear and increase in height, indicative of α-helical structure being formed as a function of TFE concentration. The results are highly similar to those observed before for rmC1 [46]. For rmC8, the results are similar to rmC1, again with somewhat lower peak heights. Multidimensional heteronuclear nuclear magnetic resonance spectroscopy also showed the formation of stable α-helices in several distinct regions of rmMBP in 30% TFE-d_2_ [48].

As far as the conformation in membrane surfaces is concerned, MBP has normally not been studied in vesicle suspensions using spectroscopic methods, due to the turbidity related to vesicle aggregation by MBP; rather, detergent micelles have often been used as membrane mimics. While the latter was also the case in the beginning of the current study, we did, indeed, explore the conditions to study MBP also in the context of vesicle samples by CD spectroscopy. We were able to measure reliable CD spectra from MBP- small unilamellar vesicle samples (Figure 5A), when reducing the protein/lipid ratio enough. While a ratio of 1/150 was still too high, samples measured at a molar ratio of 1/300 were well suitable for CD measurements. A deconvolution of the measured spectra indicates fractions of 3, 26, and 69% for helix, strand, and other structures, respectively, for the MBP sample in phosphate buffer; with lipid vesicles present, these respective ratios change to 20, 22, and 57%. Thus, the experiment clearly indicated the formation of helical structure when MBP binds to DMPG/DMPC vesicles. This result shows it is, indeed, possible to carry out CD studies on MBP when bound to vesicles, and in the future, we will be carrying out more detailed CD work with vesicle samples using different lipid compositions; such studies will also include oriented CD experiments [49].

### Analysis of the interaction between rmMBP and supported lipids by SPR

The association of MBP with lipid components plays an important role in stablizing the multilamellar structure of myelin. Membrane binding also regulates MBP binding to its ligands, and MBP is, for example, able to link actin filaments to membrane surfaces [50]. Hence, knowledge of binding of MBP to lipid systems may provide insights into intermolecular interactions of functional significance in the organization of myelin.

**Figure 6:**
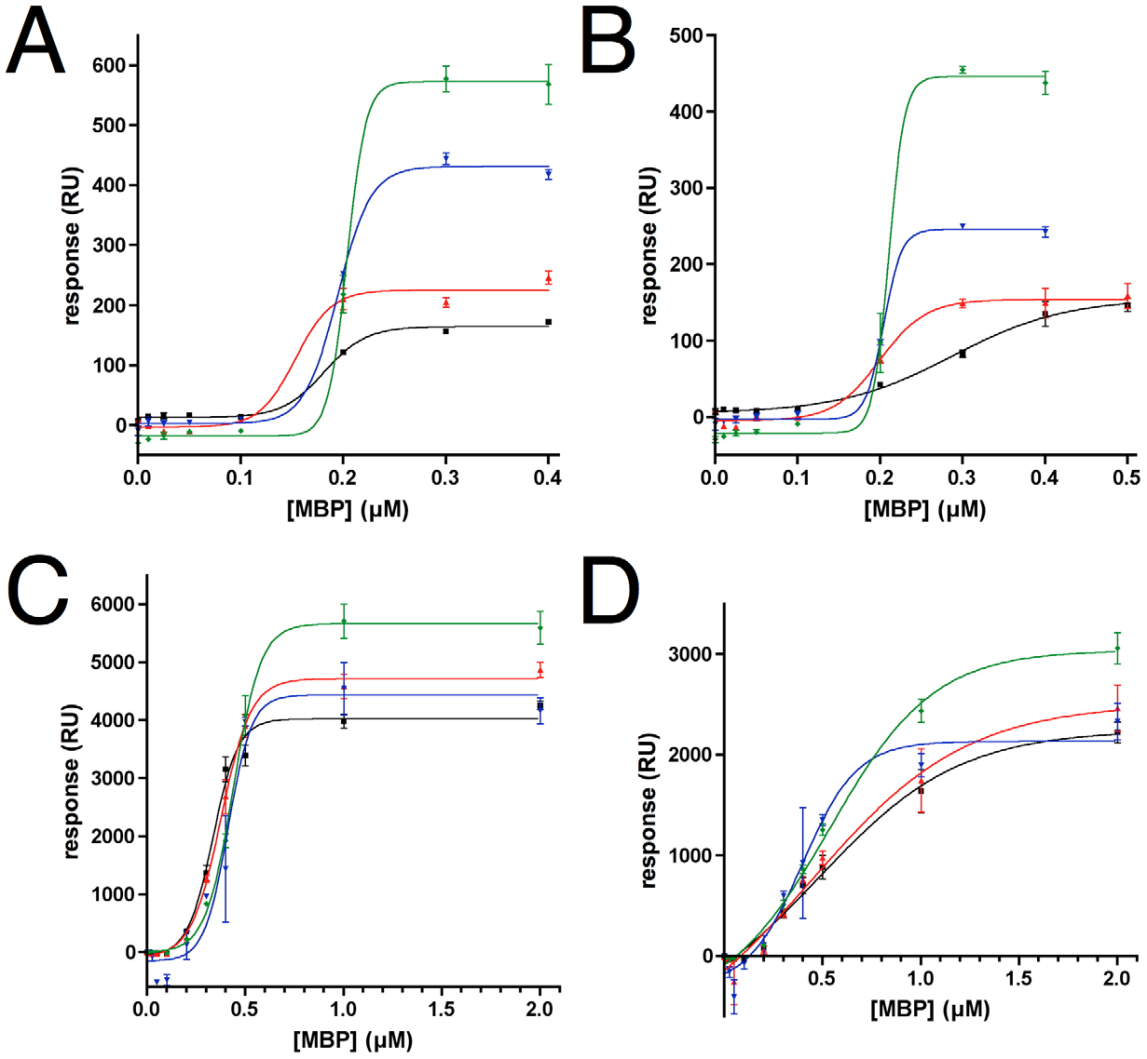
SPR analysis of MBP binding to lipidic mono- and bilayers. Black, PC; red, PC-PI; blue, PC-PIP; green, PC-PIP_2_. Curve fitting was carried out with a sigmoidal dose-response model. A. rmC1 on lipid monolayers. B. rmC8 on lipid monolayers. C. rmC1 on lipid bilayers. D. rmC8 on lipid bilayers.

Interactions between rmMBP and supported lipids with different compositions as a monolayer were investigated using SPR (Figure 6A,B). While rmC1 and rmC8 bind the monolayers in a similar manner, the affinity and total binding capacity are both slightly lower for the rmC8 isoform. The binding also shows a cooperative nature, with signal being detectable only above 0.1 µM MBP concentration, and the binding is saturated at approximately 0.3 µM protein concentration for both rmC1 and rmC8; the exception is rmC8 binding to phosphatidylcholine, where saturation only occurs at 0.5 µM (Figure 6B). With increasingly negative membrane surface charge, the binding of rmMBP increases significantly. This suggests that the interaction between MBP and lipid is largely electrostatic, and that the binding density of MBP on the lipid surface increases with negative charge in the lipid headgroups. In line with these data, it has been shown before that MBP binds negatively charged lipids with high affinity [51], [52], and that the binding can be very tight [53].

In the experiments described above, HPA chips were used to immobilize lipids; the lipids form monolayers in this case. In order to obtain more comprehensive results, L1 chips were also used (Figure 6C,D); lipid vesicles remain as bilayers on L1 chips. It can be seen that on the one hand, similar to the monolayers, more rmC1 binds onto the surface than rmC8; on the other hand, rmC8 has a clearly lower binding affinity to all the bilayers than rmC1; this is different from the results on monolayers deposited on HPA. There is binding increase of rmC1 and rmC8 with increasing negative membrane surface charge; this is, however, less drastic than on monolayers. While both mono- and bilayers show similar results for rmC1 and rmC8 binding onto lipid membrane surfaces, the effect of membrane charge seems to be smaller for the bilayer system, while the two isoforms have a larger difference in overall affinity between them in this system.

Recently, it was shown by attenuated total reflectance - Fourier transform infrared spectroscopy that Zn^2+^ could stabilize secondary structures in membrane-bound MBP, indicating a synergistic protein-membrane-cation interaction [54]. Furthermore, our SRCD studies in the presence of zinc indicated significant levels of secondary structure in MBP isolated from brain [8]. Earl *et al.* proposed that the zinc-stabilized association of MBP with brain myelin membranes might be affected by its binding to the proteolipid protein (PLP) [55]. The effect of zinc on binding of rmMBP onto a PC lipid monolayer was also assessed by SPR (Figure S1). The results further proved that Zn^2+^ can increase the association of MBP to membranes [55], at least in a certain concentration range, *i.e.* 0.1–1 mM. The binding of rmC8 to PC monolayers was increased by slightly lower zinc concentrations than that of rmC1; both isoforms completely lost binding at zinc concentrations higher than 1 mM (data not shown). We believe non-specific aggregation of MBP by high concentrations of zinc causes this behaviour. Our results clearly show that zinc increases the association of MBP with lipid monolayers, and this effect is not PLP-dependent *in vitro*.

The myelin membrane system is rigid, and possibly not fully mimicked by vesicle bilayers. Based on all the above data, we believe that both mono- and bilayer systems in SPR bring relevant information on myelin peripheral membrane protein binding to membrane surfaces.

### Different variants of the rmMBP-CaM complex by size exclusion chromatography

**Figure 7:**
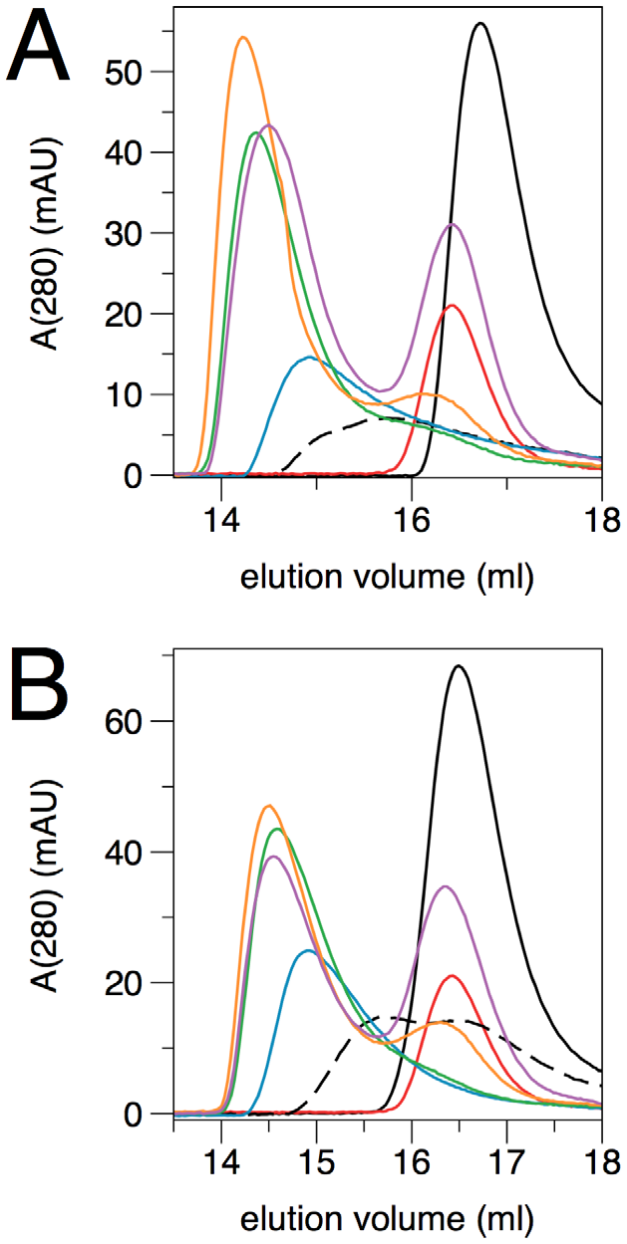
Size exclusion chromatography of MBP-CaM complexes at different stoichiometries. A. rmC1. B. rmC8. Black, MBP alone; red, CaM alone; blue, 1∶1 ratio (MBP∶CaM); green, 1∶2 ratio; orange, 1∶3 ratio; magenta, 1∶5 ratio; black dashed line, 2∶1 ratio. The elution volumes of calibration standards were as follows: 158 kDa, 12.90 ml; 75 kDa, 14.41 ml; 44 kDa, 15.20 ml; 29 kDa 16.61 ml; 13.7 kDa, 18.28 ml.

Size exclusion chromatography was used to analyze the complex formation between rmC1/rmC8 and CaM. Figure 7 shows the chromatograms at different rmMBP∶CaM ratios for rmC1 and rmC8. MBP and CaM alone elute, compared to regular globular markers, at apparent molecular weights of 29–33 kDa, reflecting their highly elongated structures, and thus, large hydrodynamic radii. The complexes with rmMBP∶CaM ratios of 1∶1 and 1∶2 obviously have different elution volumes, and there is no peak indicating the presence of uncomplexed CaM. However, the samples with rmMBP∶CaM ratios higher than 1∶2 have an elution volume close to that of the 1∶2 complex, and another separate peak appears, which exactly corresponds to CaM, not present in the complex. These results prove the formation of a stable 1∶2 complex between CaM and both rmC1and rmC8. The results also closely resemble those seen for brain MBP [8], indicating CaM complex formation by the recombinant forms of MBP is similar to proteins isolated from nervous tissue. Complex interactions and different oligomeric forms were also previously observed in gel shift assays [56]. An analysis of a 2∶1 mixture of MBP∶CaM surprisingly results in a new peak between the 1∶1 peak and the uncomplexed proteins, possibly resulting from a fast exchange between the complexed and monomeric forms. Overall, the data indicate that the MBP-CaM complex is heterogeneous, depending on the ratio of the binding partners; the different forms of the complex are stable enough to be resolved during chromatography.

### Analysis of the interaction between rmMBP and CaM by ITC

**Figure 8:**
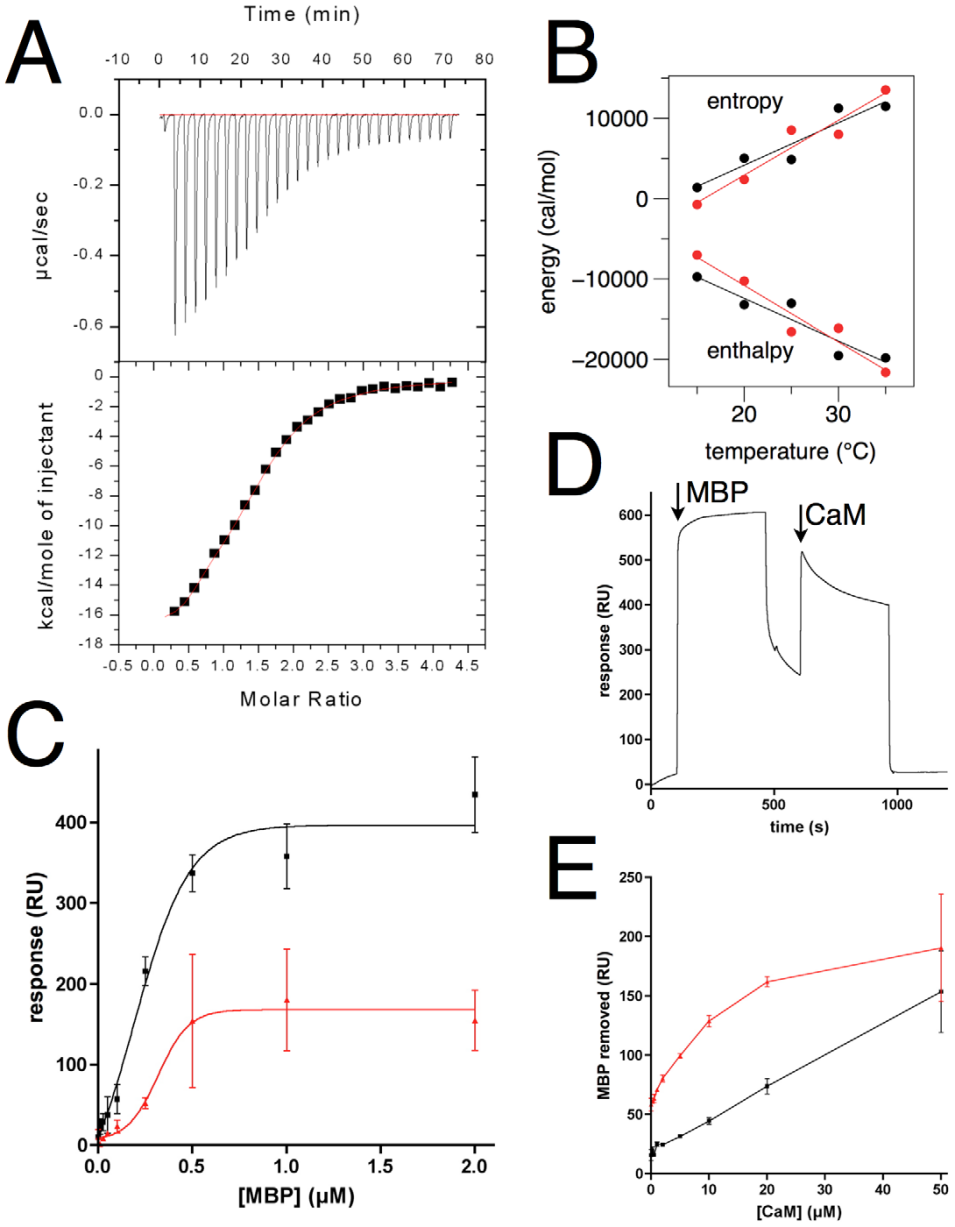
Calorimetric and SPR analysis of CaM-rmMBP interaction. A. Example of a calorimetric experiment. ITC titration of CaM into rmC1 at +30°C. B. Enthalpy and entropy as a function of temperature for the CaM-rmMBP complexes. The slope of the linear fit for the enthalpy gives an approximation of the heat capacity. rmC1, black; rmC8, red. C. SPR on MBP binding to immobilized CaM. rmC1, black; rmC8, red. D. Example of CaM stripping MBP from a PC monolayer. First, an injection of rmC8 is performed, after which 50 µM CaM is injected over the surface (arrows). It can be seen that all MBP is removed from the surface by CaM. E. The removal of MBP from the PC monolayer surface, as a function of CaM concentration. rmC1, black; rmC8, red.

MBP has been reported to interact with CaM through multiple sites [8], [57]. Here, ITC was used to study the interaction between rmMBP and CaM at various temperatures. CaM was in the syringe and rmMBP in the cell, in order to be able to separate the two binding sites during titration (Figure 8A). Previously, for MBP isolated from brain tissue, we observed two binding sites for CaM with different affinities [8]; the current ITC experiments with the recombinant MBP variants (Table 3) indicated that while the apparent stoichiometry was close to 1.5 at low temperatures, the binding curves could not be used to unequivocally distinguish the high- and low-affinity binding sites. Thus, a one-site model was used for ITC data fitting allowing the stoichiometry to float; in general, a two-site model fit the data slightly better than a one-site model, but we chose not to add parameters for no clear reason just to improve the fit. With increasing temperature, the apparent stoichiometry decreased. One possible explanation for this observation would be a near-zero enthalpy for the lower-affinity site, coupled with a rather small difference in affinity between the two sites.

**Table 3:** Calorimetric data on the MBP-CaM interaction.

Sample	Temperature (°C)	n	K_d_ (µM)
rmC1	15	1.5	0.4±0.04
	20	1.5	0.8±0.03
	25	1.9	1.0±0.07
	30	1.4	1.1±0.06
	35	0.9	1.2±0.07
rmC8	15	1.5	1.2±0.19
	20	1.3	1.3±0.08
	25	1.2	1.3±0.14
	30	1.0	1.4±0.06
	35	0.8	1.9±0.11

The data were obtained by fitting a model with one binding site, and thus, represent the overall energetics of the interaction. For the enthalpy and entropy data, see Figure 8B.

Δ*H* was plotted against temperature to estimate the change in the constant pressure heat capacity (*ΔC_p_*) for rmMBP being titrated by CaM (Figure 8B). The total *ΔC_p_* for the rmC1 and rmC8 forms of MBP binding to CaM was determined to be −530 and −680 cal/molK, respectively. The observed heat capacity is related to the buried solvent-accessible surface area upon binding. The values observed for the recombinant protein provide further evidence that MBP-CaM binding does not involve the collapse of CaM around its target sites, as the collapse of CaM around a single site generally corresponds to a heat capacity of −800 cal/molK [58]. In our previous study [8], using MBP from porcine and bovine brain, we detected affinities of approximately 10 nM and 0.1–1 µM for the two binding sites; the heat capacity for each site was approximately −200 cal/molK. The differences may be explained by the slightly different buffer conditions employed in the current and earlier studies. Taking all data together, the overall mode of binding remains apparently similar for the brain and recombinant forms of MBP. On the other hand, the values observed here also indicate that the rmC1 and rmC8 isoforms interact with CaM very similarly. It is, however, possible that their CaM regulation is different, due to the differences in their relative affinities towards other ligands, such as membrane surfaces.

### Analysis of interaction between rmMBP and CaM by SPR

CaM was covalently immobilized onto a CM5 chip, and the binding of rmMBP to CaM was investigated by SPR (Figure 8C). For both rmC1 and rmC8, the data are indicative of binding to immobilized CaM. rmC1 appears to have a slightly higher affinity and binding capacity than rmC8 in this experiment. The estimated K_d_ values are 0.2 and 0.3 µM for rmC1 and rmC8, respectively, which is similar, but slightly weaker, to the SPR experiments carried out on the brain MBP before [8]. The interaction between rmMBP and CaM was also investigated in a reverse experiment, by immobilizing rmC1 and rmC8 on a Ni-NTA chip *via* their His_6_-tag, and CaM was injected to flow through the chip. In this case, rmC1 has a higher affinity for CaM than rmC8, but the experiment is complicated by the fact that higher concentrations of CaM remove MBP from the surface (data not shown), and the data were not analyzed in detail.

Furthermore, the effect of CaM on binding of rmMBP to an immobilized lipid monolayer was assessed by SPR. The results showed that CaM can strip off rmMBP from the lipid monolayer surface, which causes the binding signal to decrease (Figure 8D,E). Similarly, it was found earlier that CaM decreased binding of MBP on PIP_2_ lipid vesicles [59]. Furthermore, CaM causes the dissociation of the MBP-actin complex from lipid vesicles [50]. For rmC1, with increasing CaM concentration, the binding signal diminished linearly in the investigated concentration range of CaM (Figure 8E). However, for rmC8, the binding decreased drastically already at lower concentrations of CaM, and all the rmC8 was removed from the supported lipids by 50 µM CaM (Figure 8D). The result implies, in accordance with the separately performed lipid and CaM-binding studies above, that the difference between the affinity towards CaM and towards the PC monolayer is greater for rmC8 than for rmC1. The main CaM-binding site of MBP is close to the C-terminus [7], [8], [57], in an area which has been shown to be poorly bound to a membrane in the rmC8 isoform [60]. During our current study, rmC8 was also shown to be, more readily than rmC1, dissociated from membranes by CaM [60]. Thus, MBP is another protein, for which CaM seems to be able to regulate the interactions between basic segments in the protein and acidic lipid bilayer surfaces [61]. The current bulk of data indicates that while the C8 form of MBP is able to bind to membranes, it may be more solvent-exposed and easier to dissociate from the membrane than the C1 form; this has implications for myelin biology, since the C8 form is known to be enriched in infants and in aggressive demyelination [62], [63].

### Nucleotide binding by MBP

**Figure 9:**
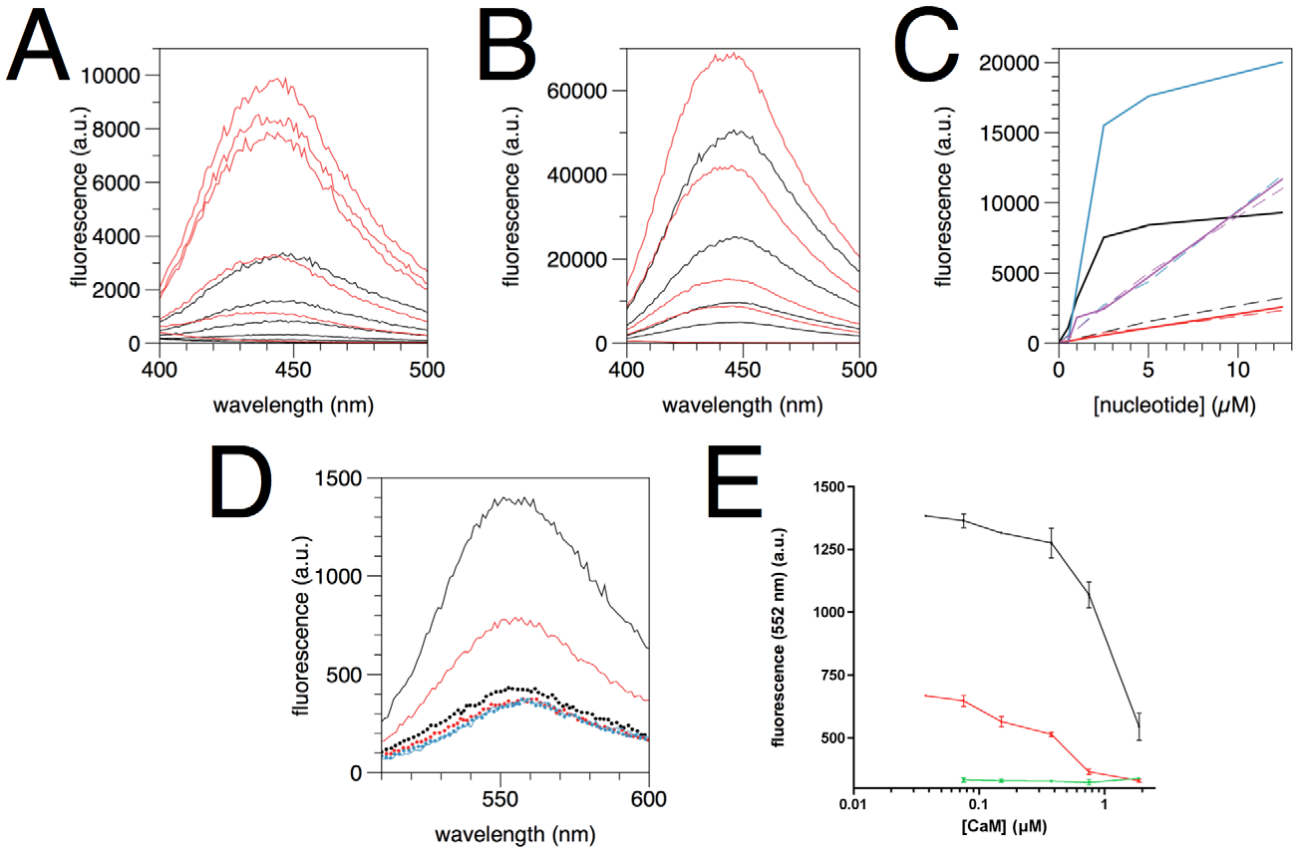
Binding of fluorescent nucleotide analogues by MBP. A. Binding of mant-ATP by rmC1. The curves correspond to different concentrations of mant-ATP (top to bottom: 12.5, 5, 2.5, 1, 0.5, and 0 µM) in the presence (red) and absence (black) of rmC1. Excitation was carried out at 280 nm, and thus, the measured signal is the sign of FRET *via* tryptophan fluorescence. B. The same samples (excluding the highest concentration) measured by excitation at 355 nm. MBP binding causes a slight increase in fluorescence and a small shift of the peak to the left. C. A comparison of different mant-labelled nucleotides, measured at 448 nm through excitation at 280 nm. Black, mant-ATP; red, mant-AMP; blue, mant-GTP; magenta, mant-GTP +7.5 µM CaM. The dashed lines correspond to samples without rmC1, and the solid lines to samples with 0.25 µM rmC1. D. Binding of 5 µM TNP-ATP. Dotted lines are samples with 7.5 µM CaM. Black, rmC1; red, rmC8; blue, no MBP. E. The effect of CaM on TNP-ATP binding, as measured by emission at 552 nm at different CaM concentrations. Black, rmC1; red, rmC8; green, no MBP.

As a further tool to analyze the structure-function properties of MBP, we used fluorescently labeled nucleotides in interaction assays. Previously, covalent labeling of MBP by azido-GTP was reported *in vitro* [64]; the relevance of this finding is still unclear. By using mant-labelled nucleotides (Figure 9A–C), it became evident that while both mant-ATP and mant-GTP bound to rmC1, mant-AMP did not; CaM abolished the binding of mant-GTP. Effects on mant fluorescence were observed when exciting both at 280 and 355 nm. Excitation at 280 nm leads to FRET through the MBP Trp residue, indicating its proximity to the bound nucleotide. We believe this interaction is, due to the high positive charge of MBP, mainly electrostatic in nature, and in a way, mimics binding to the membrane surface. Since MBP has no folded globular 3D structure, a specific binding of tri- but not mononucleotides in a functional sense seems unlikely.

Another fluorescent nucleotide analogue, TNP-ATP, bound to both rmC1 and rmC8 (Figure 9D). When CaM was added, the TNP-ATP fluorescence dramatically decreased and eventually dropped to background levels (Figure 9E). CaM alone had no effect on TNP-ATP fluorescence, and thus, what we observe is the specific removal of TNP-ATP from MBP by CaM. Similarly to the membrane-binding assays, rmC8 is less effective in TNP-ATP binding, and is more easily dissociated from the nucleotide by CaM. While there may be little relevance for the observed nucleotide binding in the biological function of MBP, the assay can be used as a further tool to study the structure and interactions of MBP.

### Conclusions

Structurally, rmC1 and rmC8 are highly similar in the absence of ligands, being essentially fully unfolded in aqueous buffer. Structural differences are only evident when binding to micelles is observed. Also, quite surprisingly, their binding modes to CaM resemble closely each other. On the other hand, rmC1 and rmC8 behave differently in binding to membrane mimics, in the case of both lipid monolayers and bilayered vesicles. When bound to membrane surfaces or nucleoside triphosphate analogues, rmC8 can be more easily dissociated from the complex by CaM than rmC1. It is likely that this difference relates to the mutation of two Arg residues (157 and 168) near the C terminus in rmC8, as the C terminal region has been suggested to play roles in both membrane and CaM binding. Thus, we provide further evidence at the structural level that arginine loss and deimination may significantly influence the structure and functional properties of rmMBP.

Interestingly, CaM is able to strip MBP from a membrane monolayer; the difference between rmC1 and rmC8 relates to the lower affinity of rmC8 towards the membrane surface. Thus, while the affinity of CaM towards modified forms of MBP may not be significantly lower, it can regulate their function differently from the wild-type unmodified protein, due to the differential affinities of MBP isoforms towards other ligands, mainly membrane surfaces. Taken together, the interaction of MBP with membranes is significantly affected by its structure and charge, the lipid composition of the membrane, and the interactions of MBP with ligands, including calmodulin and zinc ions. The interplay of all these factors will contribute to the biological task of MBP, the maintenance of the tight apposition between two cytoplasmic leaflets of the myelin membrane multilayer.

## Supporting Information

Figure S1 The effect of zinc on the association of rmC1 (black) and rmC8 (red) onto immobilized PC monolayers.
(JPG)
