# Supplementary figures and images for: Charge Isomers of Myelin Basic Protein: Structure and Interactions with Membranes, Nucleotide Analogues, and Calmodulin
Source: PLoS One. 2011 May 25;6(5):e19915. doi: 10.1371/journal.pone.0019915 (PMC3102069; doi:10.1371/journal.pone.0019915)

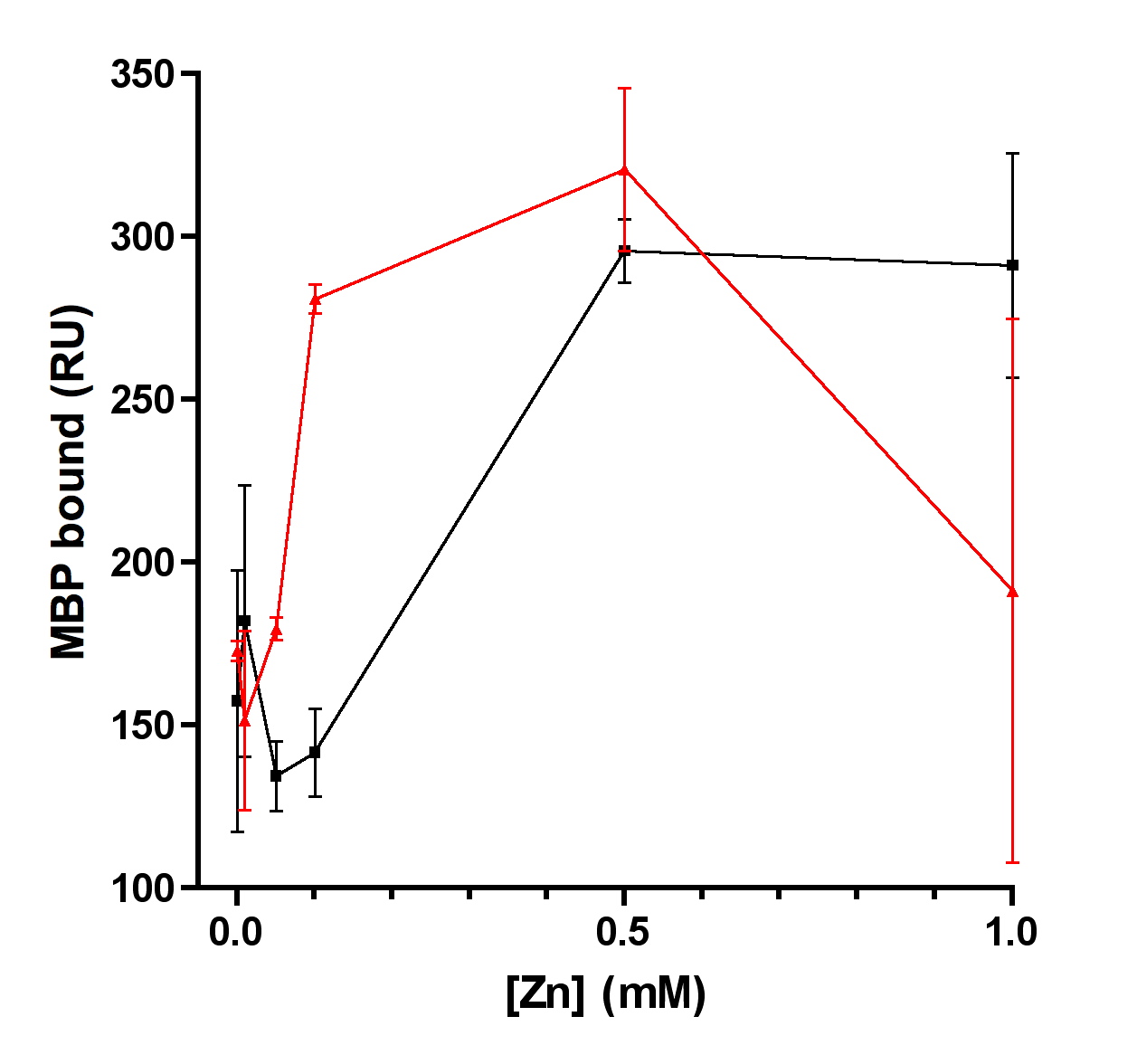

Supplement: Figure S1 — The effect of zinc on the association of rmC1 (black) and rmC8 (red) onto immobilized PC monolayers. (JPG) [file pone.0019915.s001.jpg]
